# Concurrent Effects of Plyometric Interval Training Implemented in Physical Education Lessons on Adolescent Power and Endurance: An Analysis of Responder Prevalence

**DOI:** 10.3390/sports13010015

**Published:** 2025-01-09

**Authors:** Dawid Koźlenia, Katarzyna Kochan-Jacheć, Jarosław Domaradzki

**Affiliations:** Faculty of Physical Education and Sport, Wroclaw University of Health and Sport Sciences, I.J. Paderewskiego 35, 51-612 Wroclaw, Poland; katarzyna.kochan-jachec@awf.wroc.pl (K.K.-J.); jaroslaw.domaradzki@awf.wroc.pl (J.D.)

**Keywords:** secondary school, physical fitness, youth, health, school-based setting, plyometric exercises, power, endurance, aerobic, anaerobic

## Abstract

Schools provide an ideal setting for enhancing adolescents’ health and fitness. Short-term intensive interventions are particularly relevant, but the effectiveness of plyometric exercises in jointly improving aerobic and anaerobic performance remains uncertain. This study aimed to evaluate the effects of plyometric-based training, in the form of interval workouts during physical education lessons, on power and endurance in adolescents. A total of 87 boys and 95 girls (aged 14–15 years) participated in an 8-week intervention, performing plyometric exercises twice weekly during physical education classes. The analyzed parameters were jump height (JH) measured by countermovement jump (CMJ) and distance covered during multistage fitness test (MFT). The results showed significant improvements among boys participating in the JH experiment and the distance covered in the MFT (*p* < 0.05); thus, the effect sizes were relatively small (ES < 0.3). Also, the prevalence of positive responders was more common for boys than girls; however, the difference was statistically insignificant (*p* = 0.09). These findings suggest that plyometric training has the potential for improving physical fitness, even in the context of developing opposing physical abilities, particularly in boys. However, the effects varied across individuals and were generally small, highlighting the need to optimize the intervention to achieve more pronounced results tailored to individual characteristics.

## 1. Introduction

In the context of adolescent health, body composition and cardiovascular and metabolic parameters are often emphasized, with many studies focusing on these aspects [1,2,3]. This focus is appropriate, since obesity and hypertension in childhood and adolescence significantly increase prematurely in adulthood [4]. However, it should not be forgotten that health also encompasses physical fitness [5,6], and adolescence is a critical period for developing physical fitness, which can have long-term benefits in adulthood [7].

Physical education (PE) lessons provide a natural setting for implementing various forms of physical activity to enhance physical performance [8]. In recent years, numerous authors have demonstrated that various physical activity programs introduced into physical education programs have favorable effects on health and performance [9,10]. Standard PE programs require the inclusion of substantial physical activity, but the short duration of lessons (typically 45 min) may not be sufficient to promote specific adaptations. Therefore, there is a need to implement short time interventions that address this issue without negatively impacting the PE curriculum [11,12].

Various exercise protocols have been explored in PE classes, yet no consensus exists on the optimal approach. The adaptability of these programs offers wide-ranging possibilities [13,14]. Achieving wide-ranging effects on physical fitness concurrently is also desirable. Even the development of opposite anaerobic and aerobic abilities is possible; thus, this concept is explored mainly in adults and athletes [15]. There is a gap in the field considering the joint development of power and endurance in adolescents. The topic is relevant, as (previously mentioned) the time of physical education lessons is limited and requires maximal effectiveness. In this context, implementing plyometric activities in the form of high-intensity interval training (HIIT) can be particularly relevant. It was shown that using the interval method is time-saving and effective in training focused on developing both anaerobic and aerobic abilities [15]. However, there is a lack of studies that explore this issue among adolescents. Some promising results were provided by Racil et al. [16], where HIIT with plyometric exercises was effective among young females with obesity in improving their metabolic status, but effects on physical fitness have not been checked to date.

Plyometric exercises are based on the stretch-shortening cycle [17]. When training youth and untrained individuals, it is recommended to perform low-intensity exercises with a higher number of repetitions. This approach helps to develop movement proficiency and build tissue capacity for handling higher loads. Such exercises serve as an excellent introduction for individuals unfamiliar with plyometric training for adolescents [18]. In the context of this study, this approach allows for a greater number of repetitions in a shorter period, resulting in more work performed and a greater stimulation of the cardiovascular system. Thus, concurrent improvements in both anaerobic and aerobic fitness can be expected [15,19]. A plyometric-focused approach aims to perform rapid repetitions to increase total work performed [20]. These explosive movements engage fast-twitch muscle fibers, potentially enhancing muscle power and anaerobic performance due to higher workloads in less time. Plyometric training—characterized by quick transitions between muscle lengthening and shortening—has been shown to improve motor functions, strength, and endurance in youth [21]. When combined with HIIT, it effectively engages both the nervous and muscular systems, leading to gains in strength, power, and endurance [16,22,23]. Therefore, plyometric HIIT, involving more movements and greater muscle engagement, enhances training effects and meets the criteria for a brief intervention fitted into the physical education lesson. Many studies conducted on various youth populations demonstrate improvements in jump performance following plyometric training. However, there is a lack of research within the physical education context, along with a limited consideration of its effects on aerobic capacity [24]. A study by Andrade et al. [25] showed positive concurrent effects on both anaerobic and aerobic abilities after plyometric-based interventions in adult participants. Regarding this, the implementation of this approach in adolescents during physical education lessons seems to be relevant. Moreover, plyometric exercises have shown potential not only for enhancing physical fitness but also for addressing health challenges such as obesity in children [16,26,27,28]. Previous studies have demonstrated its effectiveness in improving body composition, metabolic health, and overall physical well-being in youth populations [16,26,27,28]. This underscores the broader applicability of plyometric HIIT beyond athletic performance.

The aim of this study is to examine the concurrent effects of interval plyometric-based training conducted during physical education lessons on the development of power and endurance in adolescents. Specifically, this research seeks to evaluate how incorporating plyometric exercises within a structured school curriculum impacts physical performance metrics related to jump height and sustained endurance in school-aged individuals. Additionally, this study aims to quantify the prevalence of adolescents who experience significant improvements in measured physical performance parameters. We hypothesize that a significant proportion of adolescents will demonstrate improvements in power and endurance following an intervention program incorporated into physical education lessons. The findings from this study can inform physical education curriculum designers, coaches, and educators about the effectiveness of integrating plyometric-based training within school settings to enhance adolescents’ physical fitness. By demonstrating improvements in both power and endurance, this approach could become a valuable addition to PE programs, helping students develop critical athletic skills and overall physical capacity. Moreover, this study provides a structured framework for implementing plyometric exercises safely and effectively in classroom environments, promoting long-term health benefits and supporting students’ physical fitness.

## 2. Materials and Methods

### 2.1. Participants

Participation was voluntary, and students could withdraw at any time. Both students and their parents or legal guardians were informed about this study’s objectives and procedures. Written informed consent was obtained from school principals, parents, and participants prior to the commencement of this study. Sample size estimation was conducted using G*Power software version 3.1 [29]. Based on the adopted study design—four study groups (boys and girls, each divided into experimental and control groups) and two repeated measurements (baseline and post-intervention)—with an effect size of 0.35, an alpha level of 0.05, and a power of 0.8 [30], the minimum required sample size was determined to be 28 participants. Participants were recruited from one secondary school. Initially, eight classes were enrolled in this project. Group assignments were made using simple cluster (class) randomization without replacement via an online tool (www.randomization.com, accessed on 16 January 2024). To minimize disruption to lesson schedules and the school’s daily operations, randomization was conducted based on the division of students into classes.

Initially, 241 students from all classes were identified. Before group allocation, several students were excluded for the following reasons: refusal to participate (n = 16), medical contraindications (n = 4), and engagement in additional sports activities within the past six months (n = 12). During the intervention, additional participants were excluded (n = 27) for missing more than 20% of physical education classes. No adverse effects from the intervention were reported. The final sample comprised 179 adolescent students.

### 2.2. Procedures

**Figure 1:**
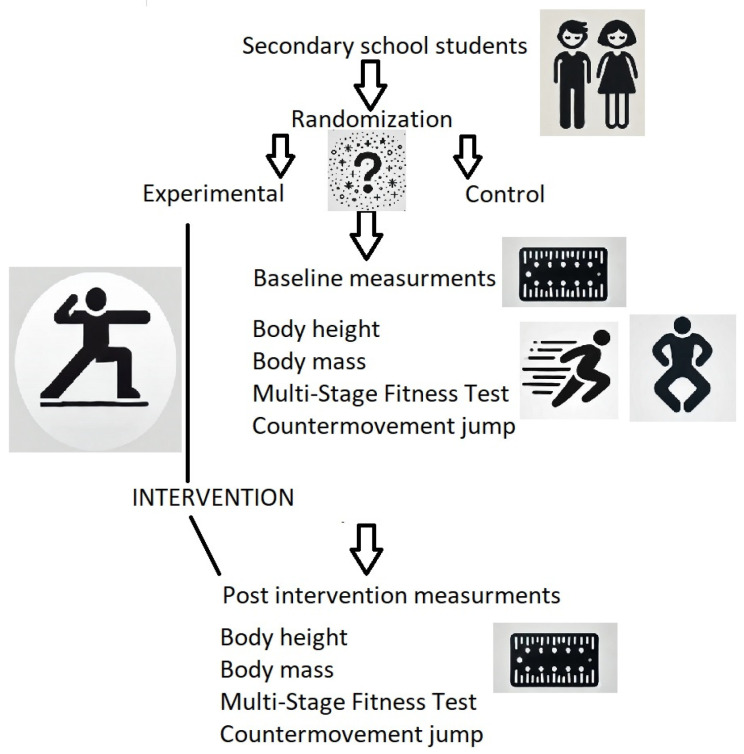
The study design.

Assessments were conducted at two time points: before the 8-week intervention (baseline) and immediately after the intervention. All measurements were performed on a single day between 8:00 a.m. and 1:00 p.m. in sports halls under standardized conditions for all groups. First, body morphology measurements were taken. Next, participants completed a warm-up, followed by the jump test and the beep test. During performance tests, participants wore T-shirts, shorts, and shoes; however, anthropometric measurements were conducted barefoot. The study design is illustrated in Figure 1.

### 2.3. Anthropometric Measurements

Body height was measured to the nearest 0.1 cm using an anthropometer (GPM Anthropological Instruments, DKSH Ltd., Zürich, Switzerland) in a standing position and barefoot, in accordance with the guidelines of the International Society for the Advancement of Kinanthropometry (ISAK) [31]. Body weight was assessed using a Tanita InnerScan V, model BC-601 (Tanita Co., Tokyo, Japan). Prior to the measurements, participants were instructed to empty their bladders, avoid excessive fluid intake, and maintain their usual breakfast routines. During the assessment, participants stood barefoot and shirtless on the scale, with heels placed on the rear electrodes, legs straight at the knees and hips, arms slightly abducted and flexed at the shoulders, elbows straight, and fingers in contact with the manual electrodes. Body mass index (BMI) was calculated as weight in kilograms divided by height in meters squared (kg/m^2^).

### 2.4. Multistage Fitness Test

To evaluate maximal heart rate and estimate aerobic capacity, the multistage fitness test (MFT) was administered. For the target audience of this study, the test/retest reliability coefficients were found to be 0.89 for children (139 boys and girls aged 6 to 16) [32,33]. The MFT requires continuous running between two lines set 20 m apart, timed to a series of recorded beeps. Participants begin running at a speed of 8.5 km/h and must turn 180° at each line. The running speed increases by 0.5 km/h every minute, as indicated by audio signals. The test continues until the participant is unable to maintain the required pace. The total distance covered, measured in meters, is analyzed.

### 2.5. Countermovement Jump Test

The countermovement jump (CMJ) test was conducted following the protocol described by Comfort et al. [34], using a Chronojump contact mat (Chronojump Bosco-system, Barcelona, Spain), whose reliability has been well-established [35]. Participants stood upright with equal weight distribution on both feet, keeping their hands placed on their hips throughout the test. They performed a rapid downward movement by bending their knees to approximately 90 degrees, immediately followed by a maximal vertical jump. Participants were instructed to execute the countermovement as quickly as possible and to maintain a consistent technique across all trials. Adopting their preferred depth of countermovement was encouraged, as this approach enhances consistency and reliability in performance, as noted by Petronijevic et al. [36]. Both feet were required to land simultaneously, and participants were instructed to ensure symmetrical take-offs and controlled landings to absorb impact. Flexion of the lower limbs during the flight phase was not permitted. Five trials were performed, with adequate rest intervals between them, and the best result was considered for analysis.

### 2.6. Intervention

A plyometric-based interval training program, following the Tabata method (20 s of work, 10 s of rest), was implemented over eight weeks, as it has previously been shown to be an effective approach for physical education lessons [37]. The intervention was conducted twice per week during physical education classes. Students had a total of three hours of physical education weekly, distributed across two days. On one of these days, students had two consecutive hours, which allowed for this specific scheduling. The eight-week duration was selected to ensure continuity of the intervention while avoiding interruptions caused by national holidays, religious observances, or school activities that could lead to missed classes. The training volume increased progressively throughout the intervention. During the first two weeks, students completed four rounds of exercises per session. This increased to six rounds in weeks three and four and to eight rounds in the final four weeks. The target workout intensity was set at 7–8 on the rating of perceived exertion (RPE) scale [11], and participants were familiarized with the RPE scale before the intervention began. Each session started with a standardized 10 min warm-up.

The intervention included the following plyometric exercises: ankle hops, burpees, high knees, shoulder taps with hand claps, butt kicks, two-leg mountain climbers, squat jumps, and alternating-leg mountain climbers. Students were instructed to perform as many repetitions as possible during each 20 s work interval. Each 20 s round focused on a single exercise, which was alternated systematically to maintain engagement and prevent stagnation. Exercises were alternated between upper and lower body movements to reduce fatigue in specific muscle groups, allowing for greater overall training intensity and ensuring variety to sustain participant motivation. This full body approach also aimed to enhance cardiovascular and respiratory system activation by engaging a larger number of muscles.

For the remainder of the physical education classes, students in the intervention group followed the standard first-year curriculum, focusing on skill development across various sports. The control group continued with the same standard physical education program throughout the intervention period. This program comprised activities aligned with the first-year curriculum, including team sports such as basketball, soccer, and volleyball, and individual activities like running and gymnastics. The focus was on skill acquisition, teamwork, and general fitness development, with no additional emphasis on plyometric training or structured interval programs.

### 2.7. Statistics

The data were presented as means, standard deviations, and 95% confidence intervals. Change values (Δ) were calculated by subtracting pre-intervention results from post-intervention results. To assess data characteristics, Shapiro–Wilk’s test was used to check normality, Levene’s test to evaluate homoscedasticity, and Mauchly’s test to assess sphericity. A three-way repeated measures ANOVA (sex × intervention × time) was utilized. Partial eta-square (pη^2^) values were calculated and interpreted as small (0.01), moderate (0.13), or large (0.26). When statistically significant differences were obtained, Tukey’s post hoc tests for varying sample sizes were conducted. Cohen’s effect sizes (ESs) were calculated and classified as small (≤0.2), moderate (≤0.5), or large (>0.5) to evaluate detailed differences [38,39]. Responders (Rs) and non-responders (NRs) were identified based on whether they showed significant improvement after the intervention. For classification based on changes in CMJ and MFT results (Δ), the typical error (TE) approach was applied, consistent with recent studies [40]. The following equation was used: TE = SDdiff/√2, where TE represents the typical error, and SDdiff denotes the standard deviation of the difference between post- and pre-intervention values. The chi-square (χ^2^) test was applied to assess whether sex differentiated the prevalence of positive effects. An alpha level of *p* < 0.05 was established for statistical significance across all tests. All calculations were performed using Statistica 13.0 software (StatSoft Poland, Krakow, Poland).

## 3. Results

Table 1 provides the descriptive statistics of the analyzed parameters, categorized by groups.

Three-way (sex × time × intervention) repeated measures ANOVA revealed that the factors of sex, intervention, and time independently and significantly affected jump height outcomes (all *p* < 0.01). Additionally, interactions between sex × time and intervention × time were statistically significant (*p* < 0.05). For the covered distance measured during the MFT, only the main effects of sex and time were statistically significant (both *p* < 0.01). No significant interaction effects were observed, although the interaction between intervention and time showed a trend (*p* = 0.07) (Table 2).

Tukey’s post hoc test for various ‘n’ revealed a statistically significant difference in jump height among boys in the experimental group when comparing baseline and post-intervention results (*p* < 0.01; ES = 0.18). Additionally, all sex differences were statistically significant at both time points (all *p* < 0.01). For changes in the covered distance during the MFT, Tukey’s post hoc test for various ‘n’ was performed. The results revealed that boys in the experimental group generally covered greater distances than girls (*p* < 0.01). Moreover, a comparison considering the time factor indicated that participants improved their MFT results after 8 weeks (*p* < 0.01).

In the final step of the analysis, the prevalence of positive responders in the experimental sample was assessed, categorizing participants as total responders (those who showed improvement in both tests), partial responders (those who showed improvement in only one test), and non-responders (Table 3). The results showed that positive changes were more prevalent among boys across all categories, while fewer boys were non-responders. However, the chi-square test indicated that sex differences in effect prevalence were not statistically significant, although a trend was observed (chi = 6.40; *p* = 0.09).

## 4. Discussion

This study demonstrated the significant potential for improving both anaerobic and aerobic abilities through the integration of plyometric exercises into physical education lessons. Specifically, boys showed significant improvements in jumping ability after the intervention, although the effect size was relatively small. In contrast, the improvements among girls were not statistically significant. Endurance differences were mainly associated with sex and time, suggesting that physical activity during physical education lessons may generally improve endurance across both sexes. Additionally, the analysis of responder prevalence revealed considerable variability in individual responses to the intervention, emphasizing the importance of tailoring programs to individual needs. While the findings are particularly promising for boys, they highlight the need for program optimization to ensure effectiveness across diverse groups.

The low-volume high-intensity intervention, in the form of interval training, emerges as a promising method for developing concurrent motor abilities [15]. Within this context, high-intensity interval training (HIIT) incorporating plyometric exercises has shown the potential for broad physical development. Previous studies have demonstrated that interval training with plyometric exercises can lead to improvements in physical fitness among female adolescents [16]. A study by Varma et al. [41] showed that plyometric training directly enhances jump height, with noticeable effects in both boys and girls, particularly among untrained individuals. This can be attributed to the specificity of jump-based exercises that target neuromuscular adaptations related to explosive power, as supported by findings from Markovic [42]. The structure of our intervention, with a 2:1 work/rest ratio, aligns with previous research by Ekstrom et al. [43], who also observed positive physical fitness development without significant sex-based differences. However, our findings diverge from these studies, as girls in our study did not show statistically significant improvements, and the prevalence of non-responders was higher among girls than boys. This discrepancy underscores the need for further investigation, particularly since other studies, such as Marta et al. [44], find no differences in power abilities between boys and girls after plyometric training during physical education lessons. Their findings further validate the utility of plyometric training in such settings for effective physical fitness development. Similarly, Kryeziu et al. [45] demonstrated that plyometric training could significantly improve speed and power in adolescent males, highlighting its potential for structured interventions. Recent research by Ma et al. [46] revealed that structured jump interval training improved jump height and multistage fitness test (MFT) performance among young female gymnasts. The lack of responsiveness among girls in our study suggests that variations in study samples, settings, or intervention designs may account for these differences. These findings emphasize the importance of optimizing program design and tailoring interventions to individual needs for maximizing their effectiveness. Endurance improvements were less pronounced. Our study did not demonstrate significant improvement in endurance after the intervention, although boys covered more distance than girls, and results improved after eight weeks. This improvement may be partially attributed to general participation in physical education, as the control groups also showed some improvement. A study by Ramirez-Campillo et al. [47] demonstrated that plyometric training in adults improved both endurance and strength, suggesting its potential for concurrent physical performance enhancements. Similarly, research with young soccer players [48] and findings by Andrade et al. [25] highlighted the dual benefits of plyometric training for endurance and power development. While these studies primarily involved adults or specific athletic groups, their results illustrate the potential of plyometric training for broad physical fitness improvement across different age groups [49]. Our results also revealed differences in responder prevalence between sexes, with boys showing more consistent positive responses. Studies such as those by Schmitz et al. [50] and Domaradzki et al. [37] highlight the sex-specific effects of HIIT, suggesting that females may respond differently due to physiological response, along with muscle fiber composition and hormonal differences [51,52]. These disparities underscore the importance of considering sex-specific factors when designing interventions, particularly in school-based settings.

This study has several limitations that warrant attention in future research. First, daily physical activity levels were not controlled. Although individuals participating in organized sports training were excluded, we did not comprehensively assess participants’ overall activity levels. Second, participant attrition led to unequal group sizes, complicating data interpretation. Third, heart rate was not continuously monitored during the intervention, limiting insights into exercise intensity and its specific effects. Future studies should address indicated limitations to enhance the reliability and applicability of findings. Using wearable devices, such as activity trackers or pedometers, alongside self-reported activity logs, can provide a more comprehensive understanding of participants’ daily movement and energy expenditure. This approach will help isolate the effects of the intervention by accounting for variations in non-exercise activities and sedentary behavior. Future studies could investigate the reasons for attrition through pre-study surveys or interviews, identifying common barriers such as scheduling issues or lack of motivation. Strategies like offering flexible scheduling, regular check-ins, or participant incentives could improve retention rates. Continuous heart rate monitoring during interventions should be implemented to better understand exercise intensity and its effects. Wearable heart rate monitors can provide real-time data, ensuring adherence to prescribed intensity levels while offering insights into variability and recovery. Exploring individual variability in responses—based on factors such as baseline fitness, maturation, or sex—could lead to more tailored and effective exercise interventions. Despite these limitations, this study also has notable strengths. We developed a safe, simple, and easily implemented training program suitable for integration into standard physical education lessons. The program requires no specialized equipment, making it broadly applicable for enhancing various physical fitness components. Additionally, a few studies have addressed the specific issues explored here, making our findings a valuable contribution to the existing literature. This study partially fills gaps in understanding the impact of plyometric training in school settings, paving the way for further research to refine and optimize such interventions.

## 5. Conclusions

The aim of this study was to evaluate the effectiveness of plyometric-based interval training, integrated into physical education lessons, in developing concurrent aspects of physical fitness. Anaerobic ability was assessed through jump height, while aerobic capacity was measured by the distance covered in the multistage fitness test (MFT). The intervention proved effective in improving jump ability among boys; however, the changes observed in girls were not statistically significant. The endurance changes were not directly related to the intervention. The impact of the intervention was nuanced, as the analysis revealed that positive effects were not highly prevalent. This finding underscores the importance of considering individual characteristics that may influence outcomes, with sex emerging as a particularly relevant factor. Despite these limitations, this study provides valuable insights that can aid physical education instructors, school administrators, and youth sports coaches in designing programs that incorporate plyometric exercises. These findings highlight the need to tailor interventions to account for individual differences, maximizing their effectiveness in school settings.

## Figures and Tables

**Table 1:** Study participants’ descriptive statistics.

Variable	Boys in the Experimental Group n = 52	Boys in the Control Group n = 35	Girls in the Experimental Group n = 51	Girls in the Control Group n = 44
	Mean ± SD (95%CI)	Mean ± SD (95%CI)	Mean ± SD (95%CI)	Mean ± SD (95%CI)
Calendar age [years]	14.54 ± 0.54 (14.39–14.69)	14.57 ± 0.78 (14.3–14.84)	14.55 ± 0.58 (14.39–14.71)	14.52 ± 0.59 (14.34–14.7)
Body height [m]	1.76 ± 0.07 (1.74–1.78)	1.78 ± 0.07 (1.75–1.8)	1.65 ± 0.06 (1.63–1.67)	1.65 ± 0.06 (1.63–1.67)
Body mass [kg]	63.25 ± 9.59 (60.58–65.92)	66.8 ± 13.74 (62.08–71.52)	57.16 ± 10.18 (54.29–60.02)	55.98 ± 8.11 (53.51–58.44)
Body mass index [kg/m^2^]	20.32 ± 2.65 (19.58–21.06)	21.02 ± 3.08 (19.96–22.08)	20.92 ± 3.41 (19.97–21.88)	20.67 ± 2.99 (19.76–21.58)
Pre-CMJ height [cm]	34.00 ± 5.06 (32.59–35.41)	32.93 ± 4.42 (31.41–34.45)	24.13 ± 3.75 (23.08–25.19)	22.34 ± 3.76 (21.2–23.49)
Post-CMJ height [cm]	34.91 ± 5.13 (33.48–36.34)	33.09 ± 4.53 (31.54–34.65)	24.58 ± 3.82 (23.5–25.65)	22.18 ± 3.76 (21.03–23.32)
Δ-CMJ height [cm]	0.91 ± 1.4 (0.52–1.31)	0.17 ± 1.13 (−0.22–0.55)	0.45 ± 1.11 (0.13–0.76)	−0.16 ± 1.01 (−0.47–0.14)
Pre-multistage fitness test [m]	1479.23 ± 403.4 (1366.92–1591.54)	1389.71 ± 420.29 (1245.34–1534.09)	772.16 ± 240.5 (704.51–839.8)	826.82 ± 249.8 (750.87–902.76)
Post-multistage fitness test [m]	1581.15 ± 403.35 (1468.86–1693.45)	1449.14 ± 475.72 (1285.73–1612.56)	838.82 ± 243.42 (770.36–907.29)	848.64 ± 294.87 (758.99–938.28)
Δ-multistage fitness test [m]	101.92 ± 189.95 (49.04–154.81)	59.43 ± 169.74 (1.12–117.74)	66.67 ± 158.48 (22.09–111.24)	21.82 ± 107.04 (−10.72–54.36)

Abbreviation: Pre—measures before the intervention; Post—measures after the intervention; Δ—difference between post- and pre-measures.

**Table 2:** Three-way repeated measures ANOVA results for countermovement jump (CMJ) and multistage fitness test (MFT) outcomes.

Effects	CMJ			MFT		
	F	*p*	pη^2^	F	*p*	pη^2^
Sex	262.57	<0.01 *	0.59	167.30	<0.01 *	0.48
Intervention	7.55	<0.01 *	0.05	0.60	0.44	0.00
Time	14.69	<0.01 *	0.08	26.98	<0.01 *	0.13
Sex × intervention	0.25	0.61	<0.01	2.00	0.16	0.01
Sex × time	5.05	0.02 *	0.03	2.30	0.13	0.01
Intervention × time	14.63	<0.01 *	0.08	3.30	0.07	0.02
Sex × intervention × time	0.21	0.69	<0.01	<0.01	0.96	<0.01

* Statistically significant difference (*p* < 0.05).

**Table 3:** Prevalence of positive changes after intervention.

Group	Boys		Girls	
	n	%	n	%
Total responders	14	26.92	9	16.66
Jump height responders	18	34.61	14	25.92
Distance responders	11	21.15	10	18.51
No-responders	9	17.30	21	38.88

## Data Availability

The raw data supporting the conclusions of this article will be made available by the authors upon request.
